# Orbital and Meningeal Metastases Secondary to Breast Adenocarcinoma: A Case Report

**DOI:** 10.7759/cureus.100821

**Published:** 2026-01-05

**Authors:** Ricardo Noguera Louzada, César Cimonari de Almeida, Pedro Lucas Machado Magalhães, Dillan Cunha Amaral, Adroaldo de Alencar Costa Filho, Marcio Penha Morterá Rodrigues

**Affiliations:** 1 Department of Ophthalmology and Otorhinolaryngology, Faculty of Medicine, Federal University of Rio de Janeiro, Rio de Janeiro, BRA; 2 Department of Neurology, University of São Paulo, São Paulo, BRA; 3 College of Medicine, Institute of Medical Education, Angra dos Reis, BRA

**Keywords:** adenocarcinoma, case report, meningeal carcinomatosis, metastasis, orbit

## Abstract

Orbital metastasis and meningeal carcinomatosis (MC) are uncommon complications of breast cancer and typically indicate advanced disease with poor prognosis. While breast cancer is the most frequent primary tumor associated with orbital metastases in women, the simultaneous occurrence of orbital metastasis and MC is exceedingly rare.

We present the case of a 51-year-old female patient diagnosed with stage IV breast adenocarcinoma, previously diagnosed with controlled HIV infection, who presented with posterior cervical pain, progressive bilateral visual loss, and left blepharoptosis. Ophthalmic examination revealed decreased visual acuity, bilateral optic disc edema, and macular exudates. Contrast-enhanced MRI demonstrated an infiltrative orbital lesion involving intraconal fat and extraocular muscles, as well as pachymeningeal thickening. Infectious etiologies were excluded. CSF analysis confirmed markedly elevated intracranial pressure and revealed malignant cells consistent with adenocarcinoma, establishing the diagnosis of MC. Histopathological and immunohistochemical studies of orbital tissue confirmed metastasis of breast origin. Despite the initiation of radiotherapy to address orbital involvement, intrathecal chemotherapy was not pursued due to the dismal prognosis. The patient’s condition deteriorated rapidly, and she died within days, reflecting the aggressive course of advanced breast cancer with leptomeningeal dissemination.

This report highlights a rare presentation of secondary intracranial hypertension due to concurrent orbital and meningeal metastasis from breast cancer. The case underscores the diagnostic value of MRI and CSF cytology, as well as the limited therapeutic options available in such advanced settings. Recognition of this presentation is important, as it is associated with high mortality and primarily requires a palliative approach.

## Introduction

Orbital metastasis is an uncommon manifestation of systemic malignancy and typically reflects advanced metastatic disease. Although several primary tumors may metastasize to the orbit, this event is rare, occurring in approximately 2-3% of patients with cancer [1-3]. A broad spectrum of tumors and pseudotumors can involve the orbit [2]; however, most ocular metastatic lesions preferentially affect the choroid. Detailed histopathological analyses, nonetheless, frequently reveal multifocal orbital involvement beyond clinically apparent lesions [4]. Among women with disseminated malignancy, breast carcinoma is the most common primary tumor associated with orbital metastases. Importantly, ocular manifestations may arise several years after the initial breast cancer diagnosis and account for nearly half of reported orbital metastatic lesions, with published series describing rates ranging from 48% to 53% [1,2,5].

Meningeal carcinomatosis (MC), also known as leptomeningeal carcinomatosis (LMC) or carcinomatous meningitis, is characterized by diffuse infiltration of malignant cells into the leptomeninges and dissemination through the cerebrospinal fluid (CSF) [6,7]. This process represents a noninfectious inflammatory condition caused by metastatic spread from extracranial primary tumors to the pia mater. Clinically, MC is associated with invasion of the leptomeninges and subarachnoid space and carries a dismal prognosis, with therapeutic options often limited to palliative care in advanced cases [8,9]. Obstruction of CSF flow and direct invasion of the brain parenchyma may further complicate the disease course [10]. Contrast-enhanced magnetic resonance imaging (MRI) is the most sensitive imaging modality for detecting MC [11], with leptomeningeal enhancement demonstrating a reported sensitivity of approximately 75% and specificity of 77% [12,13]. While MRI plays a critical role in supporting the diagnosis and localizing symptomatic involvement, particularly when CSF cytology is negative or inconclusive, definitive diagnosis of neoplastic meningitis ultimately depends on cytological identification of malignant cells within the CSF, which remains the diagnostic gold standard [14].

In this context, the simultaneous occurrence of orbital metastasis and MC represents an exceptionally rare and diagnostically challenging presentation of metastatic breast cancer. This report describes a unique case of secondary intracranial hypertension (IH) arising from the coexistence of these two metastatic processes, manifesting primarily through neuro-ophthalmic symptoms. By presenting this case, we aim to emphasize key diagnostic pitfalls related to nonspecific clinical presentations, underscore the importance of early consideration of metastatic disease in patients with a history of breast cancer and signs of raised intracranial pressure, and highlight imaging and cytological findings that may facilitate earlier recognition and appropriate management.

## Case presentation

A 51-year-old woman presented to our outpatient clinic with a seven-day history of progressive bilateral visual loss associated with posterior cervical pain. She also reported left upper eyelid drooping that developed during the same period. Her medical history was significant for HIV infection, for which she was receiving antiretroviral therapy, and stage IV breast adenocarcinoma, currently under systemic chemotherapy. No other relevant comorbidities were reported.

**Figure 1:**
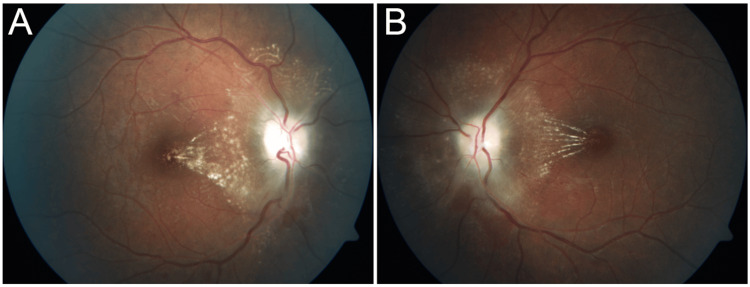
Pretreatment fundus photograph. The right (A) and left (B) eyes showing bilateral disc edema with diffuse pallor and macular edema with exudate and tortuous retinal vasculature.

At presentation, best-corrected visual acuity was 20/30 in the right eye (OD) and 20/60 in the left eye (OS). Pupillary responses, ocular motility, intraocular pressure, and anterior segment examination were within normal limits. Left-sided blepharoptosis was noted. Fundoscopic examination revealed bilateral optic disc edema with diffuse pallor, increased vascular tortuosity, disc swelling, and macular exudates in both eyes (Figures 1A-1B).

**Figure 2:**
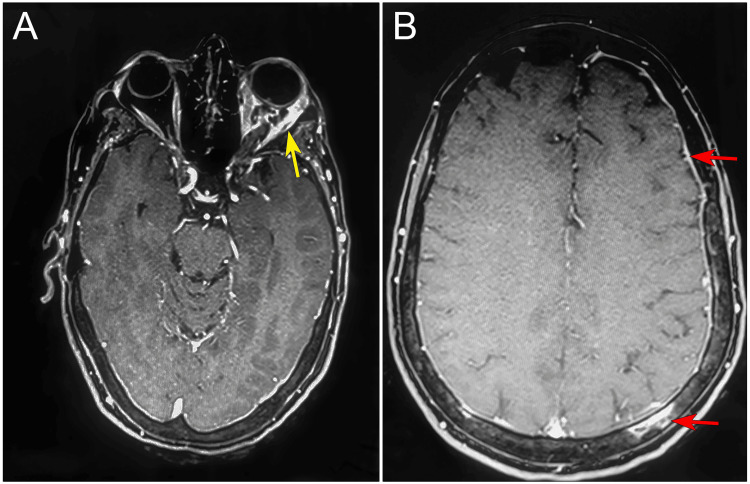
MRI imaging. (A) Axial contrast-enhanced MRI with the window set at the orbital level; the yellow arrow shows fat-suppressed orbital infiltration on the left with extensive contrast impregnation involving the extraocular musculature. (B) Axial contrast-enhanced MRI with the window set at the cranial level; the red arrows show fat suppression demonstrating metastasis in the left parietal bone and increased thickness of the dura mater, suggesting meningeal carcinomatosis.

Given the bilateral optic disc edema and neck pain, neuroimaging was promptly performed. Contrast-enhanced MRI of the brain and orbits demonstrated an infiltrative lesion in the left orbit, involving the intraconal fat and extraocular muscles, as well as diffuse pachymeningeal thickening and enhancement (Figures 2A-2B). No expansive intracranial mass lesion was identified. These findings raised suspicion for secondary IH due to leptomeningeal and orbital metastasis from breast carcinoma.

Immunological evaluation showed a CD4+ T-lymphocyte count of 416 cells/mm^3^ and an HIV viral load of 25,303 copies/mL. Despite the relatively preserved CD4 count, infectious causes of optic disc edema and meningeal involvement, including syphilis, optic neuritis, atypical toxoplasmosis, tuberculosis, and cryptococcosis, were systematically investigated and excluded.

Due to the combination of bilateral papilledema, neck pain, and MRI findings, MC was strongly suspected. Lumbar puncture revealed an opening pressure > 60 cmH_2_O, confirming severe IH. CSF cytology demonstrated malignant cells with adenocarcinoma morphology, consistent with MC.

**Figure 3:**
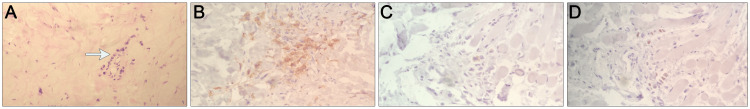
Histopathology and immunohistochemistry of orbital metastasis. (A) Hematoxylin-eosin-stained section of retro-orbital fat and medial rectus muscle showing infiltrative adenocarcinoma arranged in cords within fibrous stroma (white arrow), with abundant cytoplasm and pleomorphic nuclei (×400). (B) Immunohistochemical staining for cytokeratin 7 demonstrating diffuse cytoplasmic positivity in tumor cells (×400). (C) Immunohistochemical staining for GCDFP-15 showing positive tumor cells, supporting mammary origin (×400). (D) Higher-power view of GCDFP-15 immunoreactivity highlighting the distribution of mammary-type tumor cells (×400).

To further evaluate orbital involvement, a transconjunctival biopsy of the medial rectus muscle with intraoperative frozen section was initially performed and was negative. However, definitive histopathological examination of orbital fat and lateral rectus muscle revealed infiltration by malignant epithelial cells. Immunohistochemical staining confirmed metastatic breast adenocarcinoma, corroborating the diagnosis of MC associated with orbital metastasis (Figures 3A-3D).

Following diagnostic confirmation, the case was discussed in a multidisciplinary setting involving oncology, neurology, and ophthalmology teams. Given the patient’s advanced systemic disease, rapid neurological decline, and poor performance status (an Eastern Cooperative Oncology Group performance status of 3 (ECOG3)), aggressive interventions such as combined radiotherapy and systemic chemotherapy were considered unlikely to provide meaningful clinical benefit. One week after initiation of radiotherapy, ophthalmologic reassessment showed stable visual acuity, persistent optic disc pallor, and partial resolution of papilledema. Unfortunately, the patient experienced rapid systemic clinical deterioration, which precluded further neuroimaging follow-up.

## Discussion

This is a case report of a patient with breast adenocarcinoma that presented with secondary IH, MC, and orbital metastasis. Although both MC and orbital metastasis can occur in breast cancer, their association in the same patient is exceedingly rare.

When leptomeninges are involved, the patient may exhibit headache, progressive loss of visual acuity, and optic disc edema [15]. HIV infection was under clinical and laboratory control, with preserved CD4 count and absence of opportunistic ocular infections. Therefore, it did not directly contribute to the patient’s ophthalmic manifestations, which were attributed to IH secondary to MC from breast cancer.

Contrast-enhanced MRI is preferred over skull computed tomography for evaluating MC due to its superior soft-tissue contrast, which allows direct visualization of leptomeningeal enhancement, thickening, and nodularity. These features are often missed on CT, particularly in early or diffuse disease. Consequently, MRI detects leptomeningeal abnormalities in approximately 60-70% of cases, while CT is mainly useful for identifying gross structural changes or complications such as hydrocephalus [16,17]. Contrast-enhanced MRI using a gadolinium-based contrast agent improves lesion conspicuity by highlighting pathological leptomeningeal enhancement, thereby facilitating detection of meningeal involvement. In this case, MRI findings were suggestive of orbital metastasis, based on the presence of diffuse meningeal thickening (Figure 3B) and an infiltrative pattern involving the intraconal fat and extraocular muscles (Figures 3A, 3C), which, in the context of advanced breast adenocarcinoma, favored metastatic disease over inflammatory or infectious etiologies. MC was confirmed by CSF cytology and by the biopsy of the orbital fat and the lateral rectus (Figure 3A) with immunohistochemistry and immunoreactivity for cytokeratin 7 (Figure 3B), GCDFP-15, and estrogen receptor, which allowed us to confirm the breast adenocarcinoma as the primary site (Figures 3C-3D). MC is a fatal complication affecting 5-10% of patients with breast cancer and is associated with high mortality, for which treatment is mainly palliative, focusing on symptom control, preservation of neurological function, and improvement of quality of life rather than curative intent [10,18].

Following diagnostic confirmation, treatment decisions were made in a multidisciplinary context involving oncology, neurology, and ophthalmology teams. Radiotherapy was selected to address orbital involvement, given its effectiveness for local disease control and the limited penetration of systemic chemotherapy into the orbital fat [2]. At the time of therapeutic decision-making, the patient exhibited rapid neurological decline and poor functional status (ECOG3), which significantly limited tolerance to aggressive interventions. In this clinical context, intrathecal chemotherapy was not pursued due to the low likelihood of meaningful neurological benefit and the high risk of treatment-related morbidity.

Despite palliative-oriented treatment, the patient’s condition deteriorated rapidly, reflecting the aggressive nature of leptomeningeal dissemination in advanced breast cancer. This case highlights the importance of recognizing MC as a potential cause of IH and orbital infiltration in patients with known malignancy, as well as the need for individualized, multidisciplinary decision-making when balancing potential benefits and burdens of therapy in advanced disease.

## Conclusions

This case underscores the diagnostic challenges posed by neuro-ophthalmic manifestations in patients with advanced breast cancer and demonstrates that orbital metastasis and MC may coexist and manifest primarily as secondary IH. The initial presentation may be misleading, as infiltrative metastatic patterns can mimic infectious or inflammatory conditions, particularly in the presence of confounding but clinically controlled comorbidities, representing a major diagnostic pitfall and a potential source of delayed investigation. In this context, contrast-enhanced MRI combined with CSF cytology and targeted histopathological analysis proved decisive for establishing the diagnosis and confirming tumor origin. Early recognition of this metastatic pattern is essential, as it directly impacts clinical decision-making, informs prognostic assessment, and guides timely palliative-focused management strategies aimed at symptom control and quality-of-life preservation.
